# Supplementary figures and images for: Key necroptotic proteins are required for Smac mimetic-mediated sensitization of cholangiocarcinoma cells to TNF-α and chemotherapeutic gemcitabine-induced necroptosis
Source: PLoS One. 2020 Jan 8;15(1):e0227454. doi: 10.1371/journal.pone.0227454 (PMC6948742; doi:10.1371/journal.pone.0227454)

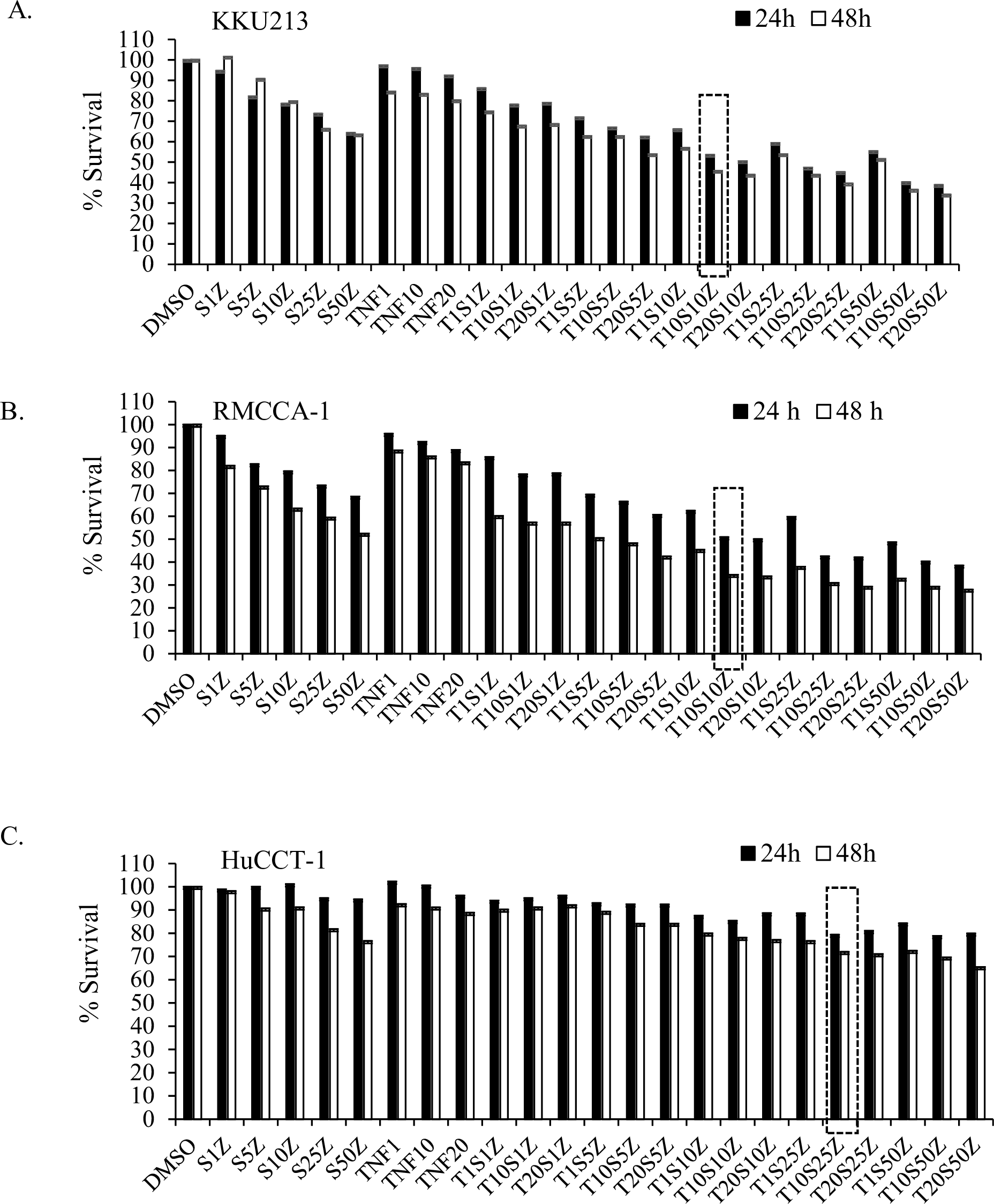

Supplement: S1 Fig — (A) KKU213 (B) RMCCA-1, and (C) HuCCT-1. Cells were pretreated with SZ (Smac mimetic, 1, 5, 10, 25, and 50 nM; zVAD-fmk, 20 μM) for 2 h, followed by treatment with T (TNF-α, 1, 10, 20 ng/ml) for 24 h and 48 h. Cell viability was determined by MTT assay. Inset indicates the concentration of TNF-α and Smac mimetic around IC50 at 24 h and was selected for further analysis. (TIF) [file pone.0227454.s001.tif]

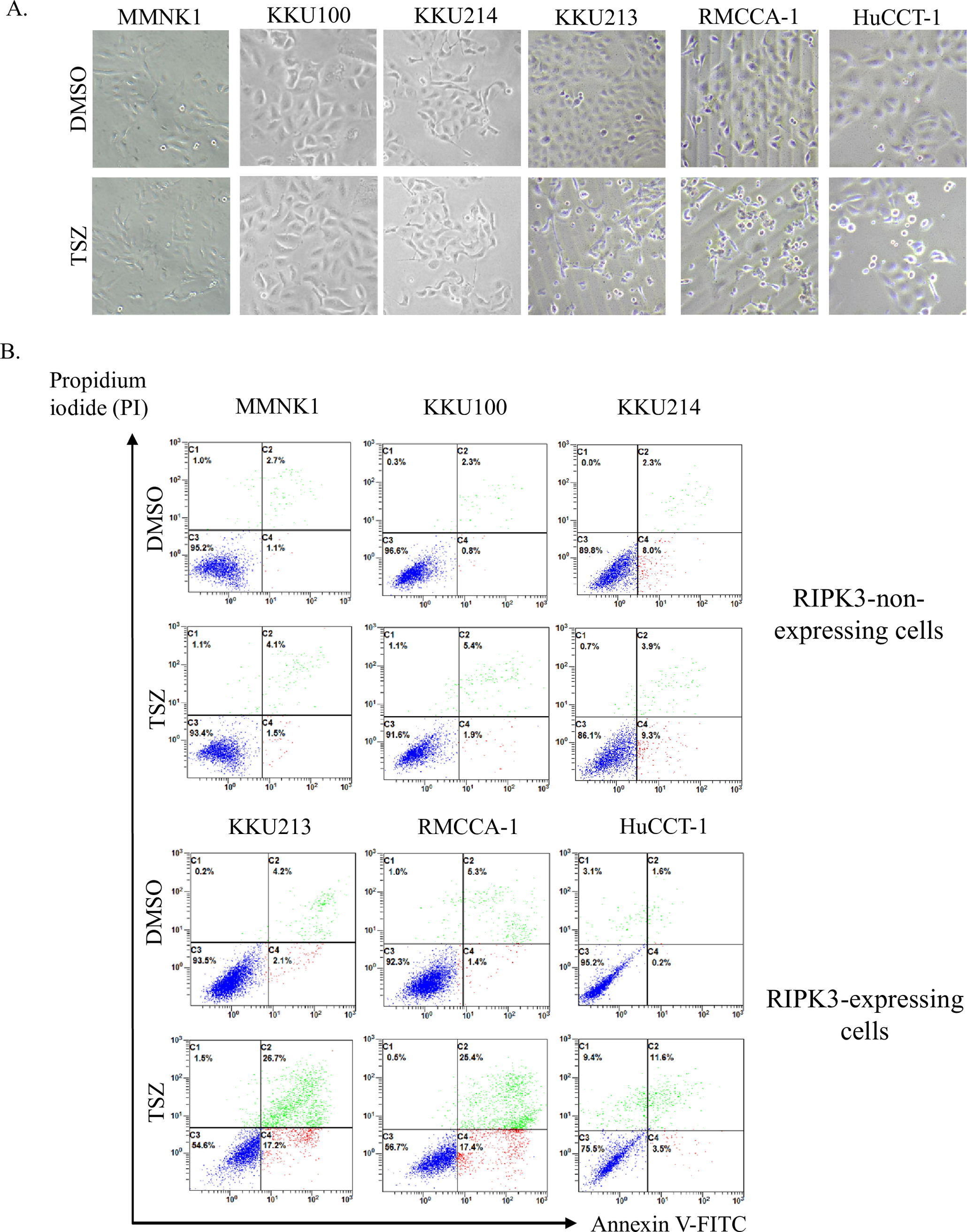

Supplement: S2 Fig — (A) Representative of cell morphology upon treatment with TNF-α/Smac mimetic in the presence of zVAD-fmk in RIPK3-deficient cells (MMNK1, KKU100, and KKU214) and RIPK3-expressing cells (KKU213, RMCCA-1, and HuCCT-1). (B) Representative of flow cytometry analysis of cells, treated as in A. (TIF) [file pone.0227454.s002.tif]

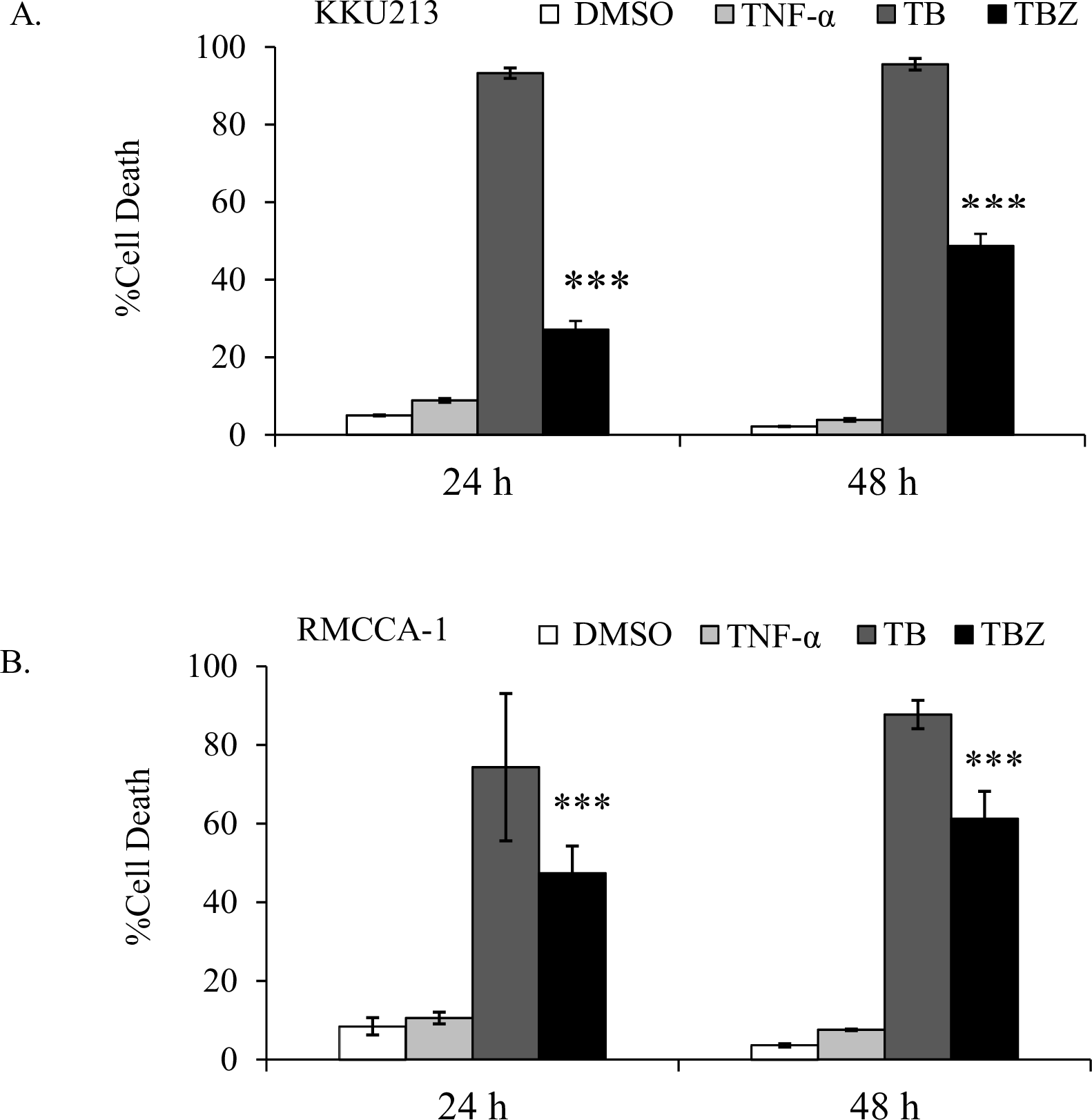

Supplement: S3 Fig — (A) KKU213 and (B) RMCCA-1 were treated with 10 ng/ml TNF-α, TNF-α and 5 μM BV6 (TB), or TNF-α and BV6 in the presence of 20 μM zVAD-fmk (TBZ) for 24 h and 48 h. Percentages of cell death (AnnexinV+/PI- and AnnexinV+/PI+) were determined by Annexin V and PI staining and flow cytometry. Data presented as mean ± S.D. of three independent experiments are shown; * p < 0.05, **p < 0.01, *** p < 0.001 (TIF) [file pone.0227454.s003.tif]

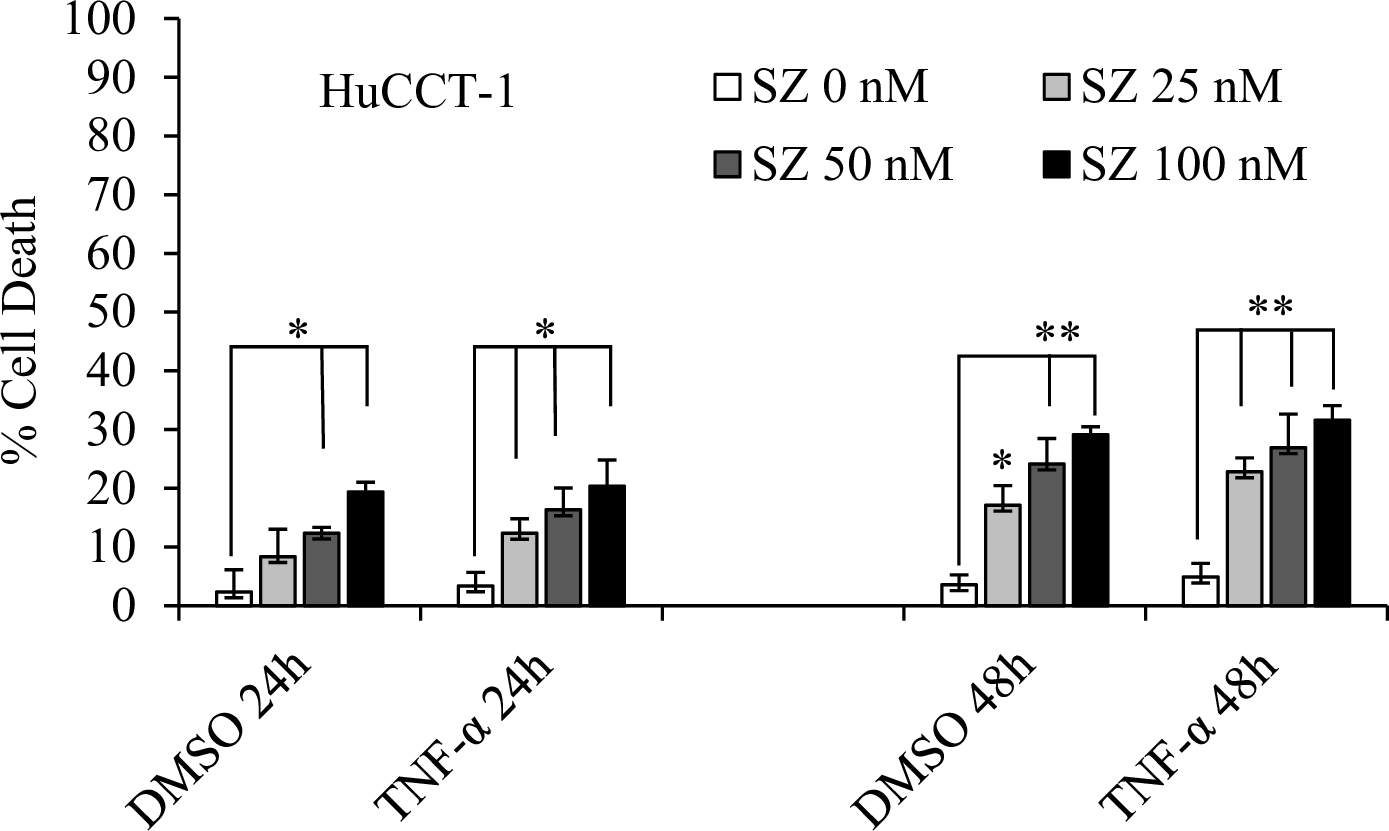

Supplement: S4 Fig — HuCCT-1 cells were treated with different concentration of Smac mimetic (S) (0 nM, 25 nM, 50 nM, 100 nM) in the presence of 20 μM zVAD-fmk (Z) with or without 10 ng/ml TNF-α for 24 h and 48 h. Percentages of cell death (AnnexinV+/PI- and AnnexinV+/PI+) were determined by Annexin V and PI staining and flow cytometry. Data presented as mean ± S.D. of three independent experiments are shown; * p < 0.05, **p < 0.01, *** p < 0.001 (TIF) [file pone.0227454.s004.tif]

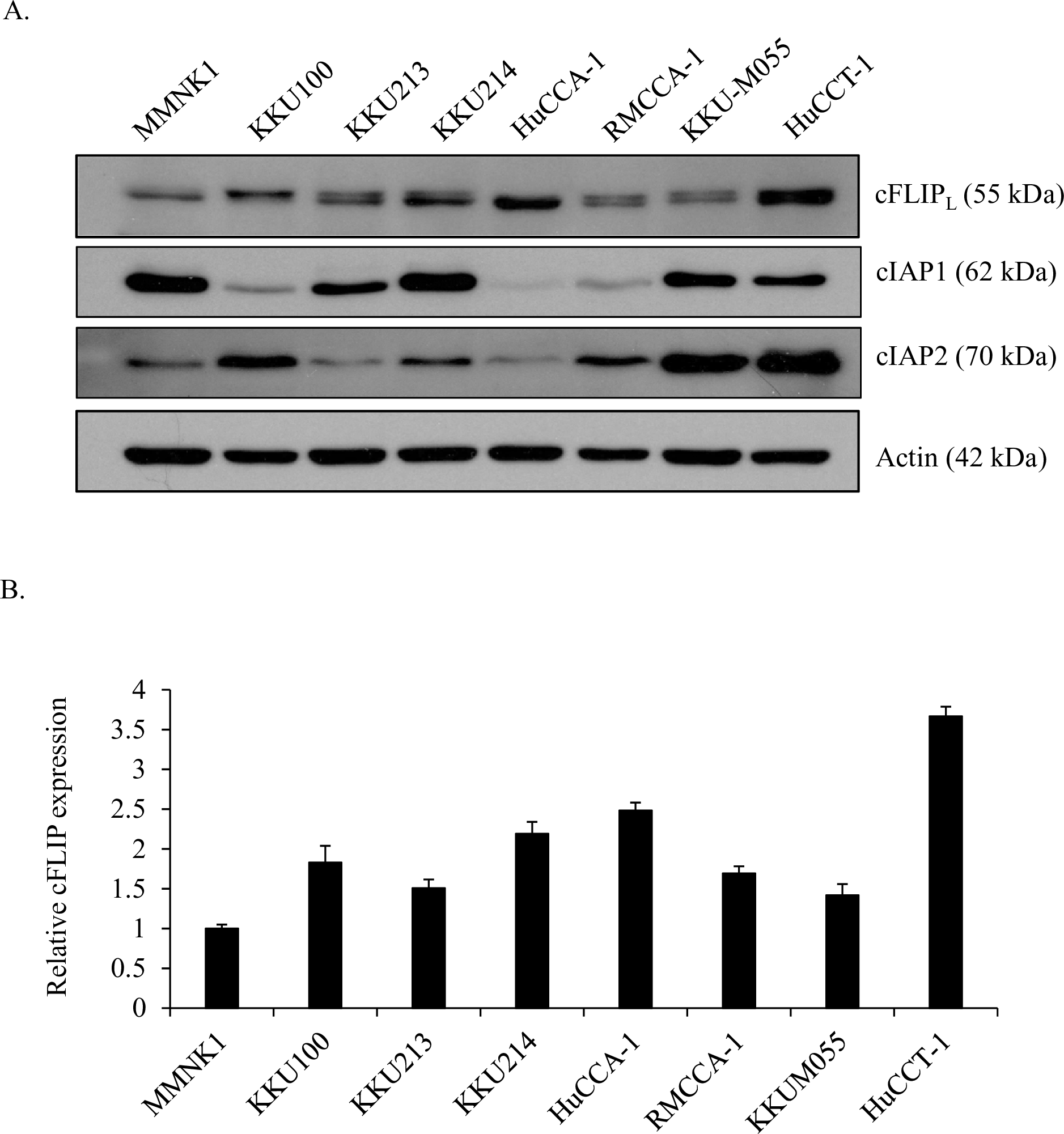

Supplement: S5 Fig — (A) Seven CCA cells and a nontumor cholangiocyte, MMNK1 cell lysates were collected and subjected to Western blot analysis. β-actin was served as loading control. (B) cFLIPL was normalized to actin protein expression, and presented as fold increase relative to MMNK1 with its mean set to 1. (TIF) [file pone.0227454.s005.tif]

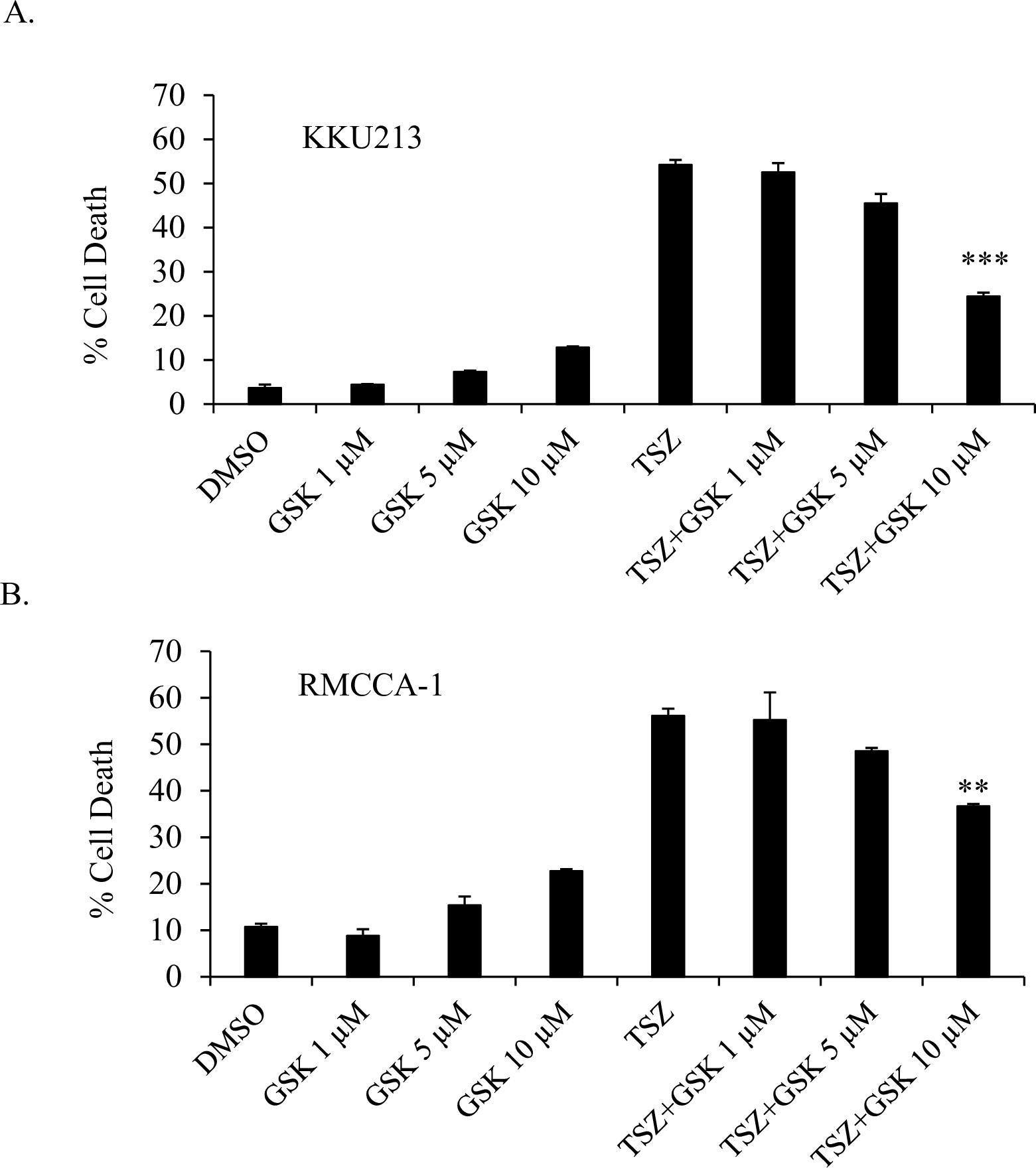

Supplement: S6 Fig — (A) KKU213 and (B) RMCCA-1 were pretreated with 1 μM, 5 μM, and 10 μM of GSK’872 and Smac mimetic/zVAD-fmk for 2 h followed by treatment with 10 ng/ml TNF-α for 24 h. Percentages of cell death were determined by Annexin V and PI staining and flow cytometry. Data presented as mean ± S.D. of three independent experiments are shown; * p < 0.05, **p < 0.01, *** p < 0.001 (TIF) [file pone.0227454.s006.tif]

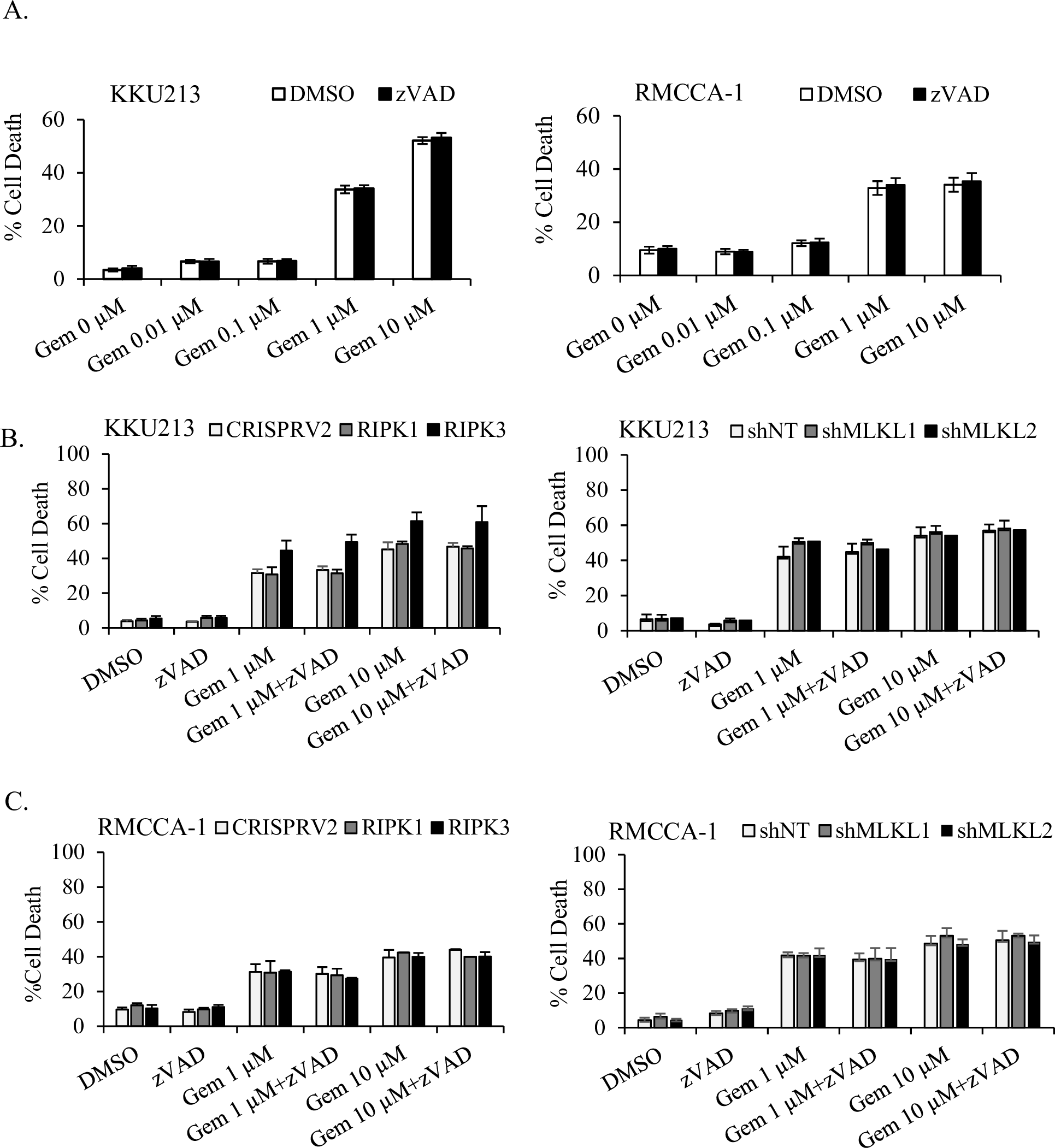

Supplement: S7 Fig — (A) KKU213 and RMCCA1 were treated with 0.01, 0.1, 1, or 10 μM gemcitabine in the presence or absence of 20 μM zVAD-fmk for 72 h (KKU213) and 48 h (RMCCA-1). RIPK1 and RIPK3 knockout or MLKL knockdown (B) KKU213 and (C) RMCCA-1 cells were treated with 1 μM or 10 μM gemcitabine in the presence or absence of 20 μM zVAD-fmk for 72 h (KKU213) and 48 h (RMCCA-1). Cell death was determined by Annexin V and PI staining and flow cytometry. Percentages of cell death presented as mean ± S.D. of three independent experiments are shown. (TIF) [file pone.0227454.s007.tif]

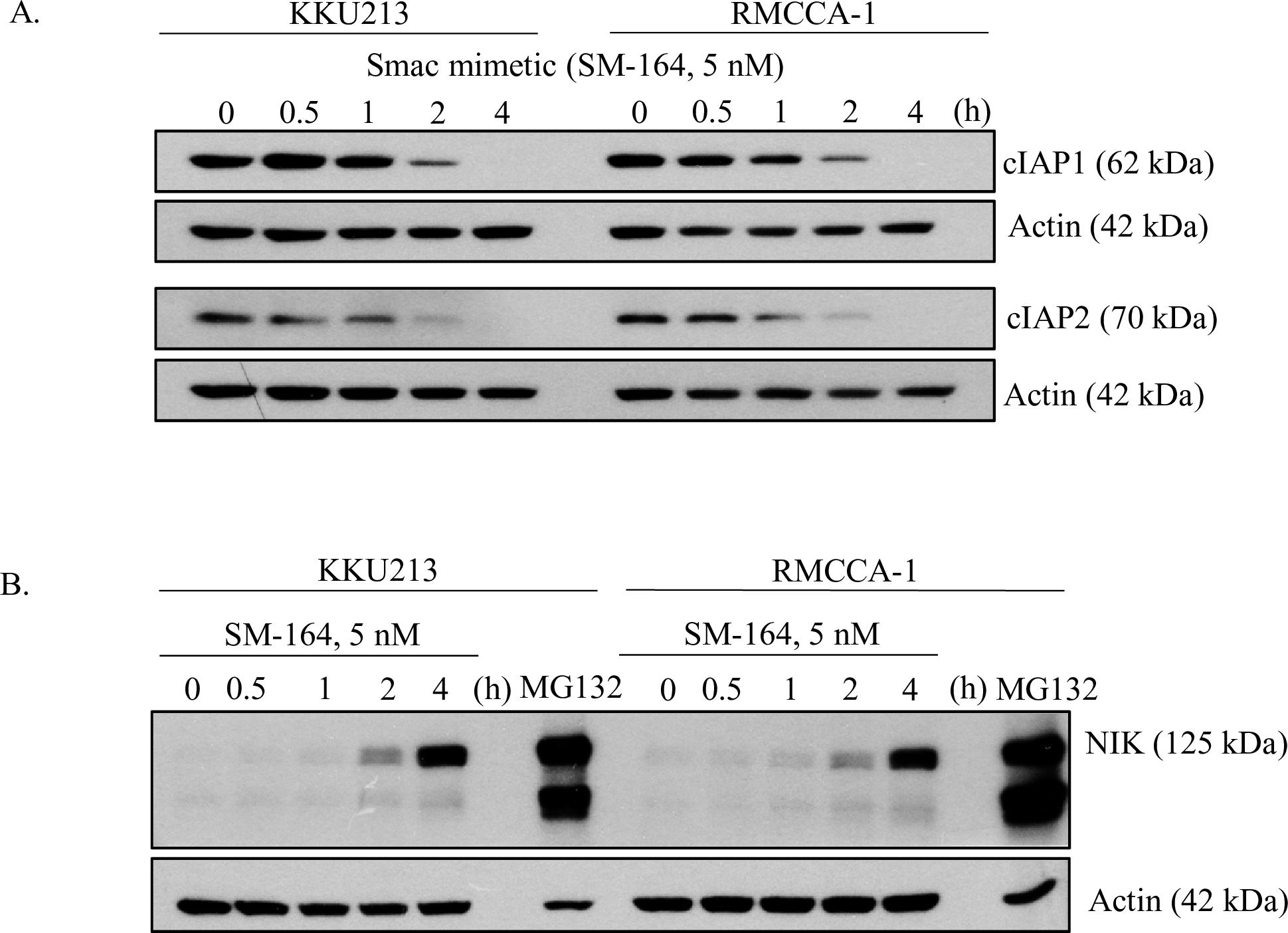

Supplement: S8 Fig — KKU213 and RMCCA-1 were treated with 5 nM Smac mimetic for indicated time points. The expression of cIAP1 and cIAP2 (A), and NIK (B) were determined by Western blot analysis. MG132 (10 μM, 6 h) was used as a positive control for NIK stabilization. β-actin served as loading control. (TIF) [file pone.0227454.s008.tif]

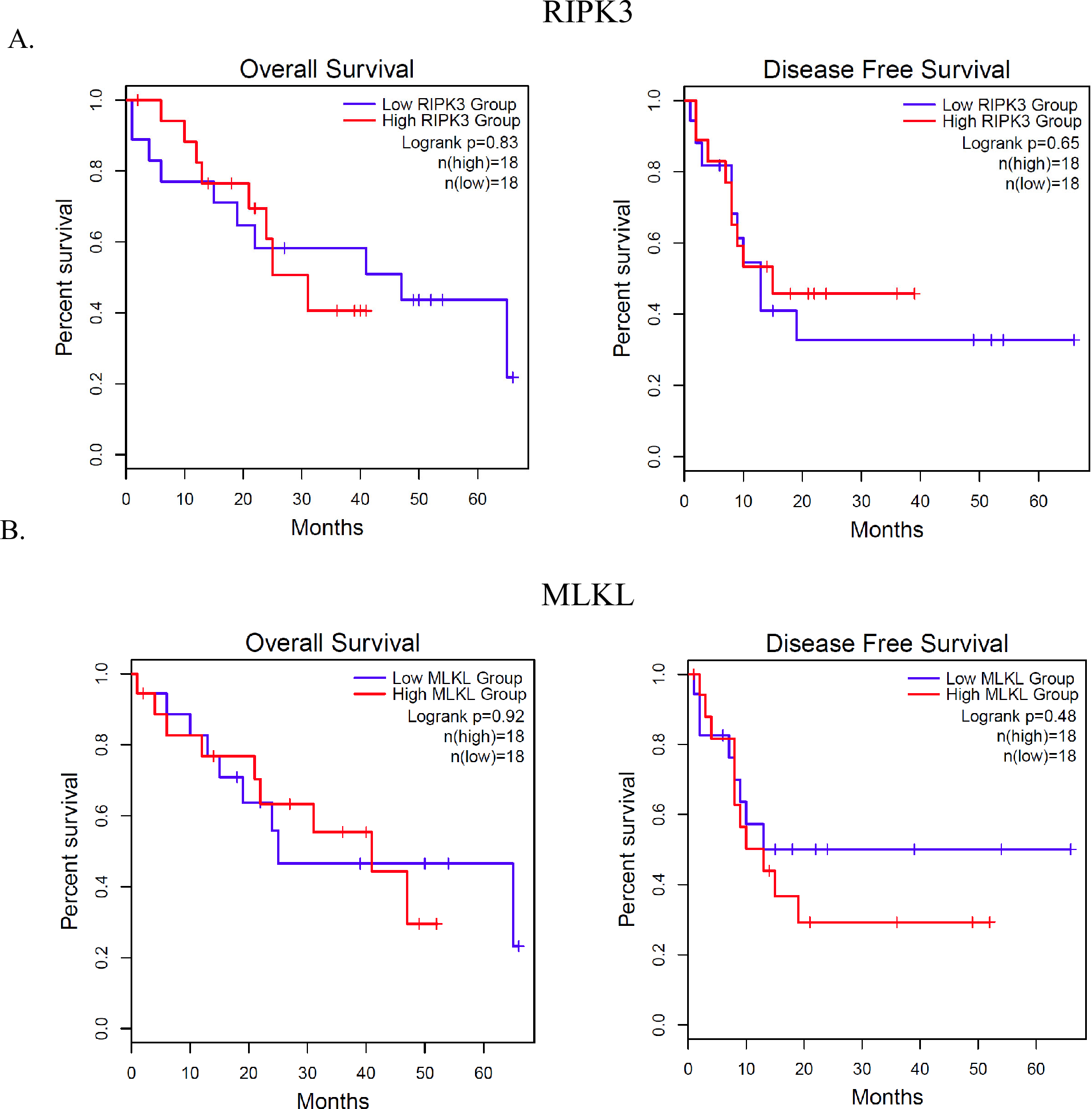

Supplement: S9 Fig — The association between overall survival or disease free survival and RIPK3 (A) or MLKL (B) expression was analyzed from GEPIA database. Samples with expression level higher than the median of TPM (transcripts of per million) are considered as the high-expression cohort (High). Samples with expression level lower than the median of TPM are considered the low-expression cohort (Low). (TIF) [file pone.0227454.s009.tif]
