# Supplementary material for: Key necroptotic proteins are required for Smac mimetic-mediated sensitization of cholangiocarcinoma cells to TNF-α and chemotherapeutic gemcitabine-induced necroptosis
Source: PLoS One. 2020 Jan 8;15(1):e0227454. doi: 10.1371/journal.pone.0227454 (PMC6948742; doi:10.1371/journal.pone.0227454)
Supplement: S1 Raw Images — Full unedited images for Figs 2A, 2B, 3A, 3C, 3D, 4D, 5A–5C, 6C. (PDF) [file pone.0227454.s010.pdf]

Full unedited images for Figure 2A and 2B

Figure 2A

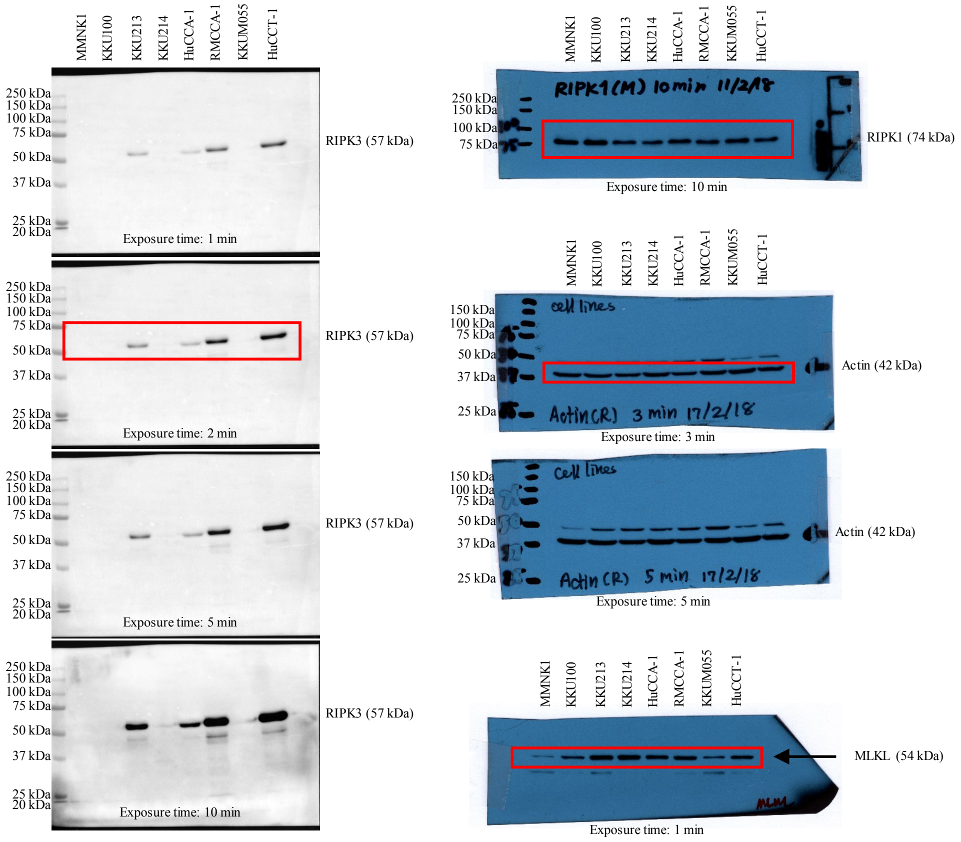

Figure 2B

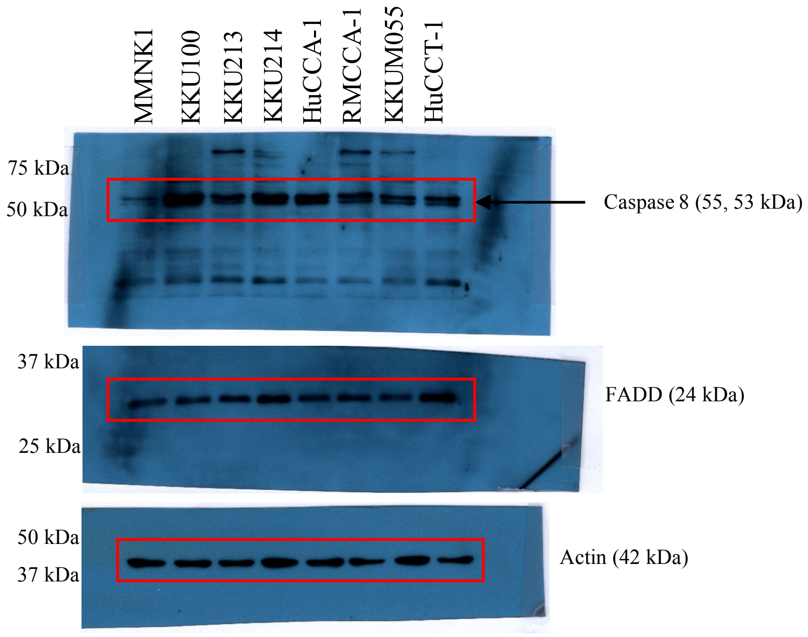

Full unedited images for Figure 3A and 3C

Figure 3A : pMLKL

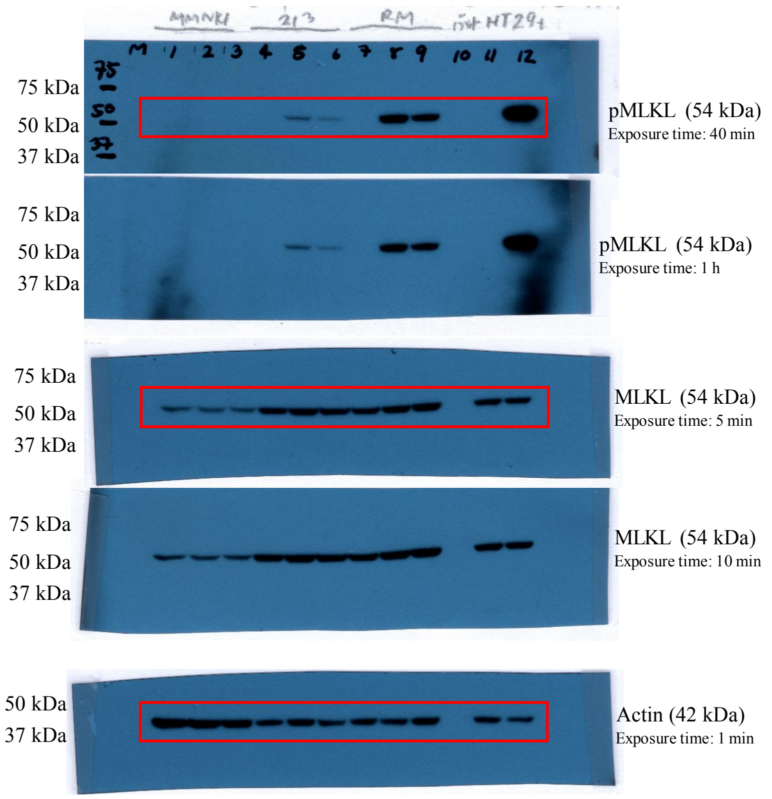

Figure 3C : KKU213 CRISPR RIPK1 and CRISPR RIPK3

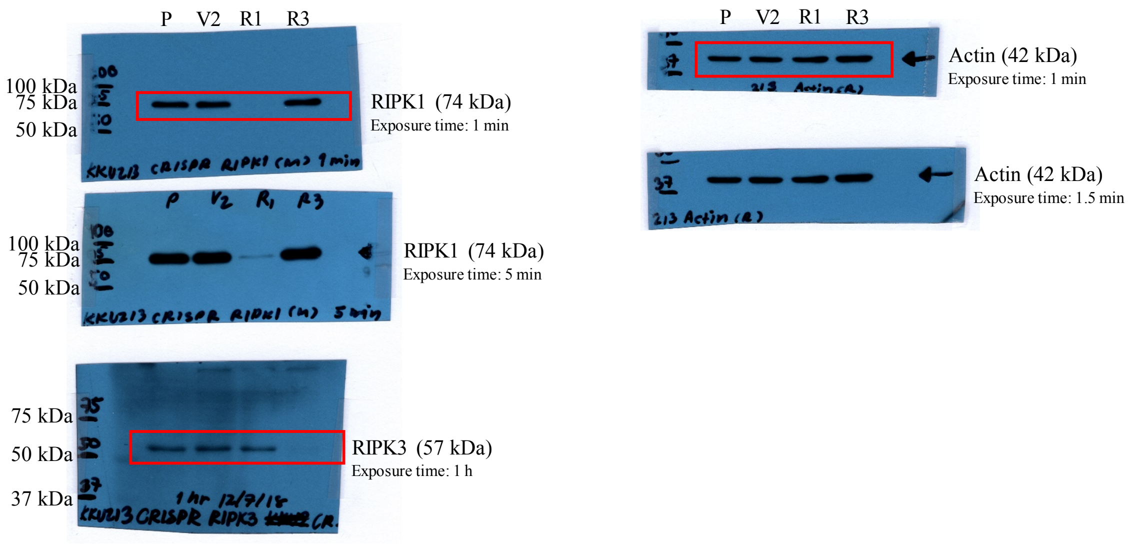

Full unedited images for Figure 3C and 3D

Figure 3C : RMCCA-1 CRISPR RIPK1 and CRISPR RIPK3

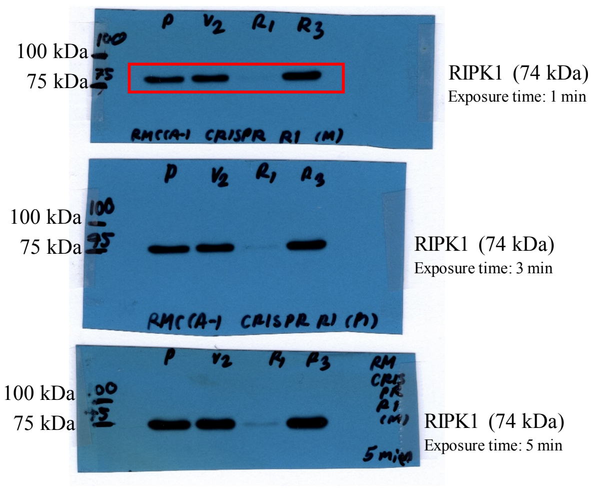

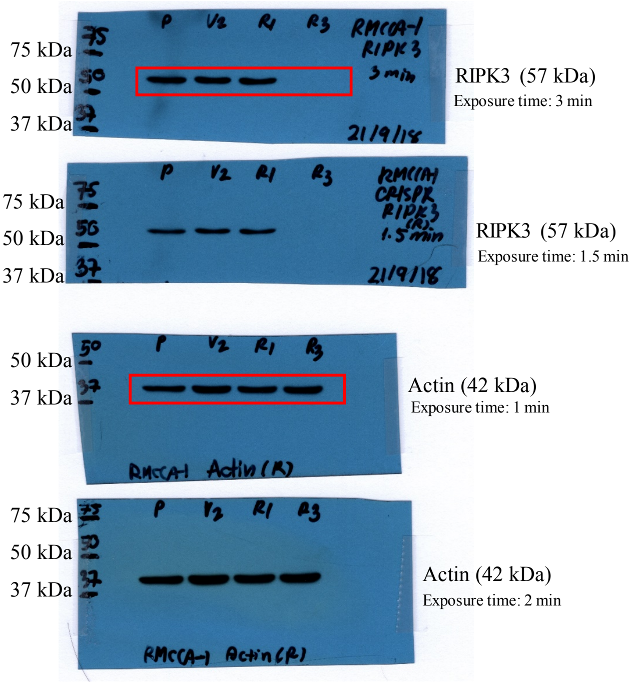

Figure 3D: KKU213 shMLKL

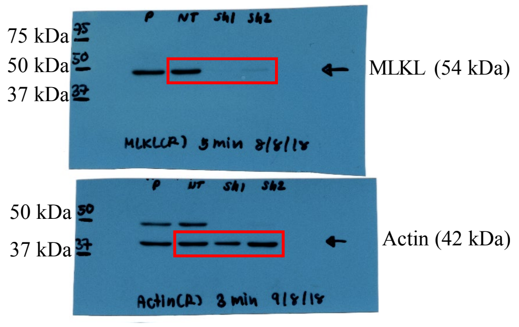

Figure 3D: RMCCA-1 shMLKL

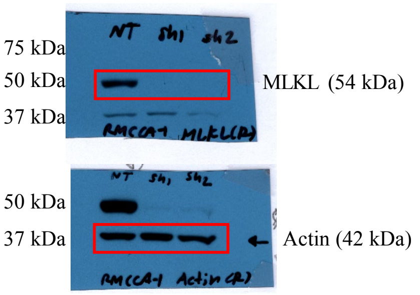

Full unedited images for Figure 4D

Figure 4D : KKU213 GSZ (pMLKL, MLKL, RIPK1, RIPK3)

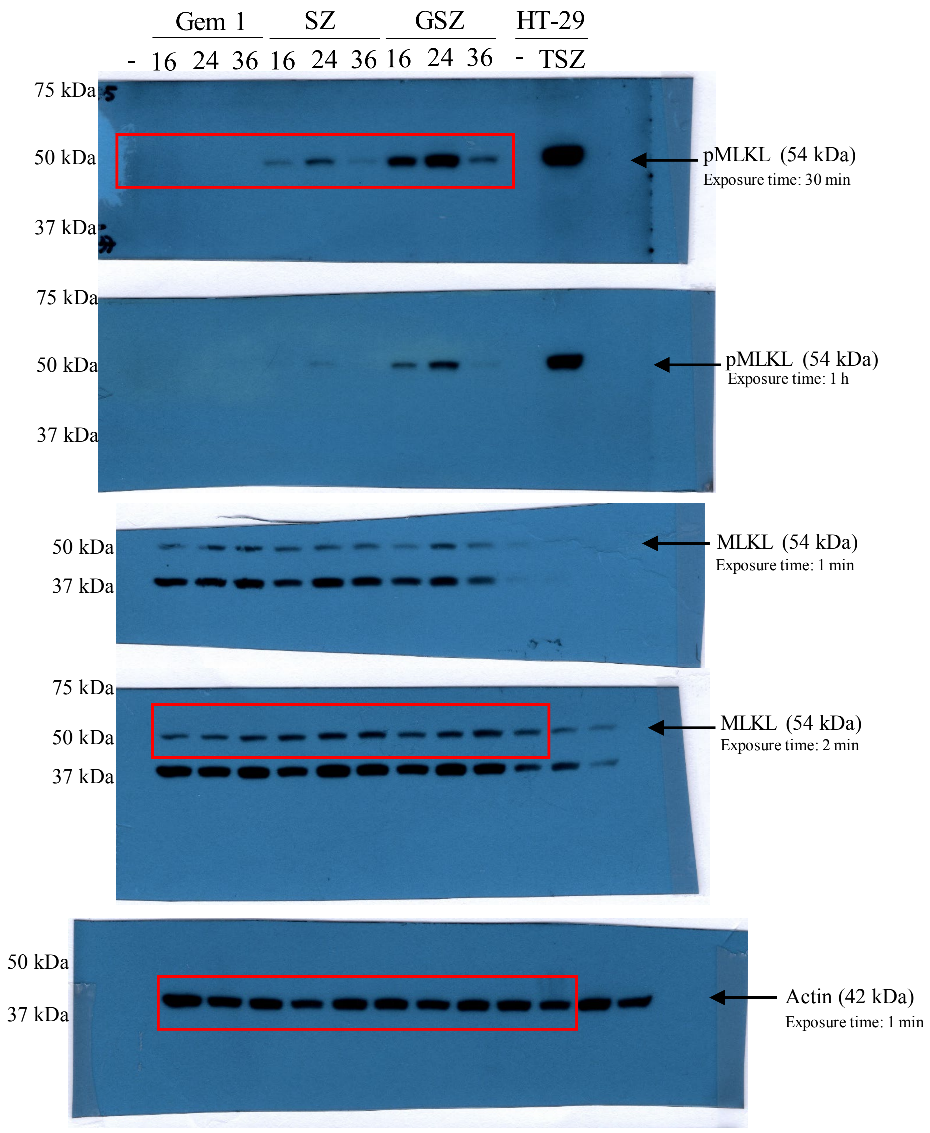

Full unedited images for Figure 4D

Figure 4D : RMCCA-1 GSZ (pMLKL, MLKL, RIPK1, RIPK3)

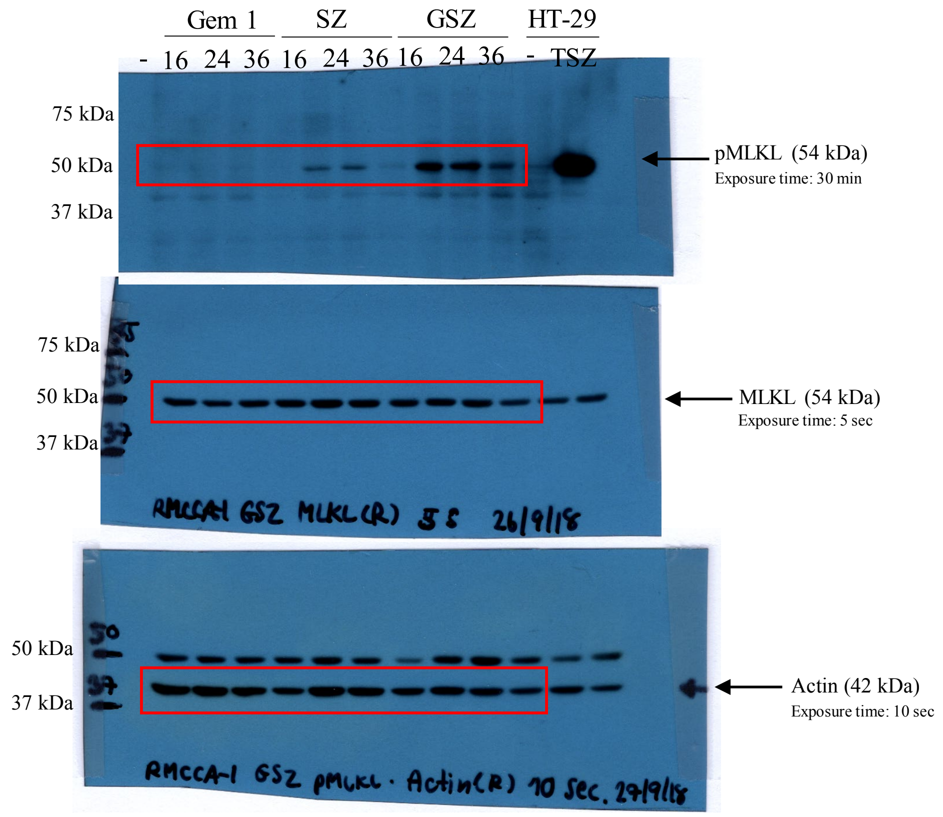

Full unedited images for Figure 5A, 5B and 5C

Figure 5A

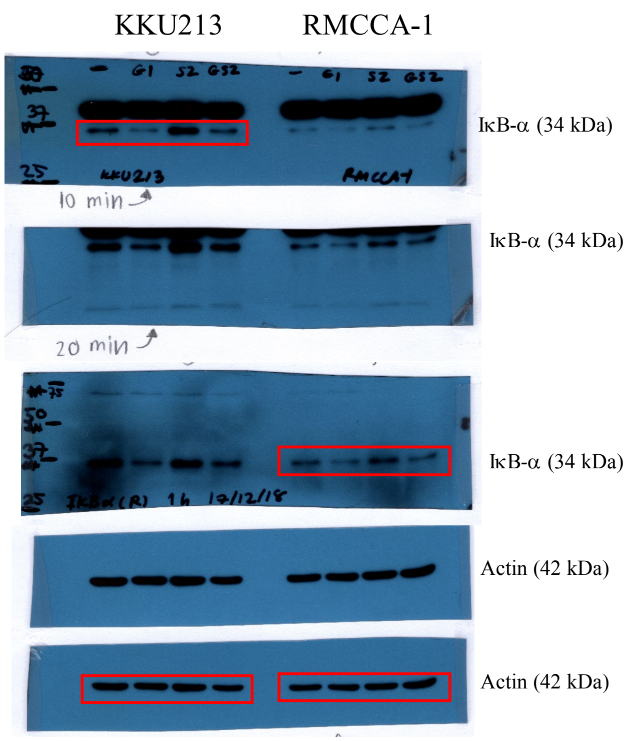

Figure 5B

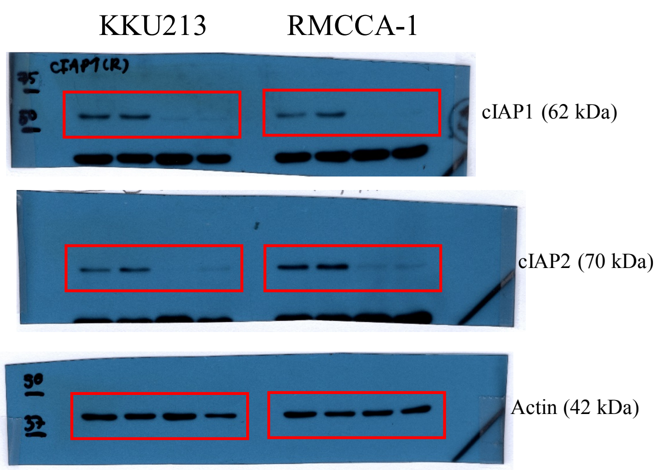

Figure 5C

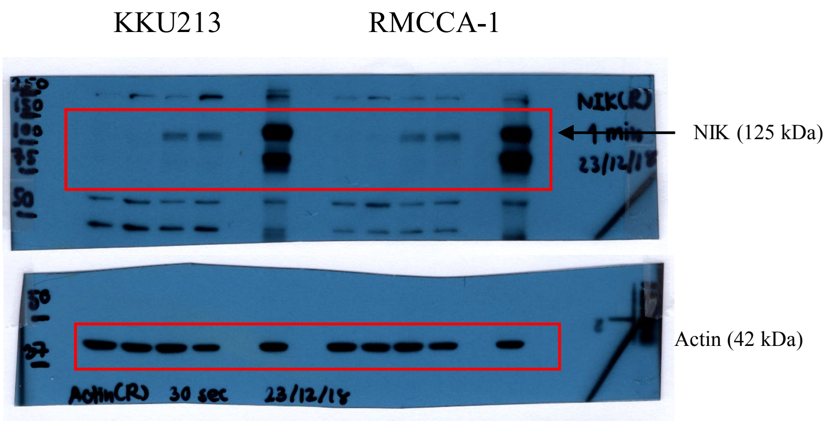

Full unedited images for Figure 6C

Figure 6C

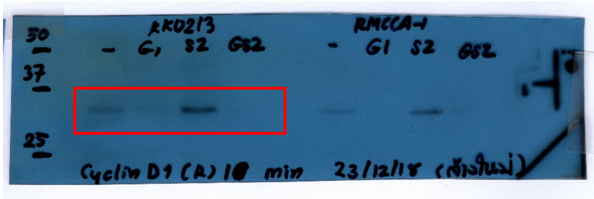

Cyclin D1 (36 kDa)

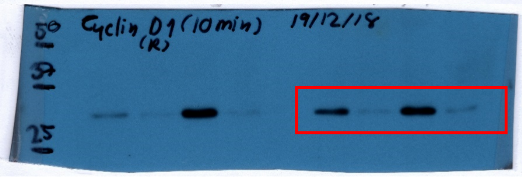

Cyclin D1 (36 kDa)

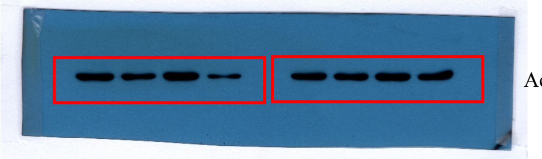

Actin (42 kDa)
